# Effects of different anesthesia methods on labor process and postpartum serum estrogen and progesterone levels in primiparas with painless labor

**DOI:** 10.1016/j.clinsp.2024.100442

**Published:** 2024-07-13

**Authors:** JunYan Liu, ChongLai Shi, Dan Wang, XiaoDong Cui, LiLi Geng, JingJing Cui, DongMei Sun, Zhuo Yin

**Affiliations:** a Department of Anesthesiology, Cangzhou Hospital of Integrated TCM-WM·HEBEI, Cangzhou City, Heibei Province, China; b Department of Anesthesiology, Yangxin People's Hospital, Huangshi City, Hubei Province, China; c Department of Military Casualty Management, General Hospital of Western Theater of Chinese People's Liberation Army, Sichuan City, Chengdu Province, China; d Department of Gynecology, Cangzhou Hospital of Integrated Chinese and Western medicine, Cangzhou City, Heibei Province, China; e Department of Cardiovascular Medicine, Yanshan People's Hospital, Cangzhou City, Hebei Province, China; f Department of Anesthesiology, DaLian Medical center For Women and Children, Dalian City, Liaoning Province, 116011, China

**Keywords:** Anesthesia method, Painless labor, Primipara, Labor process, Serum Estrogen and Progesterone

## Abstract

**Objective:**

To look into the effects of different anesthesia methods on the labor process and the expression of serum estrogen and progesterone in primiparas with painless labor.

**Methods:**

60 primiparas receiving painless labor were selected as the research objects, and they were divided into either a Spinal & Continuous epidural anesthesia group (n = 30) or a continuous epidural anesthesia group (n = 30), anesthesia is administered using the corresponding anesthesia method. The authors compared serum estrogen and progesterone, inflammatory index expression, pain degree and neonatal health status in different periods.

**Results:**

At T2 and T3, serum P, LH, FSH and E2 levels in the Spinal & Continuous epidural anesthesia group were signally lower than those in the Spinal & Continuous epidural anesthesia group (p < 0.05). Spinal & Continuous epidural anesthesia group harbored faster onset and longer duration of sensory block and motor block than the Continuous epidural anesthesia group (p < 0.05). SAS and SDS scores of the Spinal & Continuous epidural anesthesia group were clearly lower than those of the Continuous epidural anesthesia group (p < 0.05). VAS score and serum TNF-α, IL-6 levels of pregnant women in the Spinal & Continuous epidural anesthesia group were memorably lower than those in the Continuous epidural anesthesia group at T2 and T3 (p < 0.05). The total incidence of postoperative complications in the Spinal & Continuous epidural anesthesia group was distinctively lower than that in the Continuous epidural anesthesia group (p < 0.05).

**Conclusion:**

Spinal anesthesia combined with continuous epidural anesthesia has a better anesthesia effect in the painless labor of primiparas, which can effectually ameliorate the labor process and the expression of serum estrogen and progesterone.

## Highlights

• Spinal anesthesia combined with continuous epidural anesthesia has a better anesthesia effect in the painless labor of primiparas.
• Spinal anesthesia combined with continuous epidural anesthesia can effectually ameliorate the labor process.
• Spinal anesthesia combined with continuous epidural anesthesia attenuates the expression of serum estrogen and progesterone.
• Spinal anesthesia combined with continuous epidural anesthesia can be widely employed in clinical practice.

## Introduction

Painless labor is to reduce or even eliminate the pain during childbirth by diverse methods. [1] Drug-induced analgesia and non-drug analgesia are currently prevailingly utilized in clinical labor analgesia. Among them, non-drug analgesia holds less impact on the parturient and fetus and higher safety, but the analgesic effect is limited, so its clinical application rate is low. Conversely, drug-induced analgesia dominantly includes intraspinal delivery analgesia, intramuscular injection of analgesic drugs and so on, which possesses conspicuous analgesic influence and is broadly adopted in clinical applications. [2,3] Intraspinal analgesia is currently the most stable clinical analgesic approach for labor analgesia, with low concentration of anesthetic drugs, high safety, long-lasting efficacy, quick postoperative recovery, and a wide range of suitable populations as characteristics. [4] Nevertheless, some pregnant women with intraspinal analgesia suffer from adverse reactions such as hypotension, headache, and nerve damage after delivery, having a serious negative impact on the recovery of postpartum physical fitness. [5,6]

Serum P, LH, FSH, and E2 are the leading serum estrogen and progesterone indicators in the human body. Barak Y et al. [7] have corroborated that excessive reduction of serum P, LH, FSH, and E2 during and after delivery of pregnant women may lead to adverse reactions such as postpartum anxiety and depression. During the process of childbirth, the physiological changes in the body will undergo major changes. During pregnancy, the level of neuroendocrine activity in pregnant women is relatively high. Estrogen, progesterone, and thyroid hormone are usually expressed in a high state, and such hormones can play a part in modulating genes and metabolic levels through the neurotransmitter in the brain, promote the sensitivity and activity of the central nervous system, and ultimately promote a smooth pregnancy. [8,9] Ku C W et al. have evidenced in research that varying anesthesia methods will influence the recovery of estrogen and progesterone levels of pregnant women after painless labor, and eventually affect the physical health and mental health of pregnant women after delivery. [10] Epidural anesthesia and spinal anesthesia are both pervasively employed intraspinal anesthesia methods for pregnant women undergoing painless labor. The former principally injects medicine into the epidural space, while the latter primarily injects medicine into the subarachnoid space through the lumbar intervertebral space. [11,12] Currently, there are few clinical studies comparing the application value of epidural anesthesia and spinal anesthesia in painless labor of pregnant women and their influence on estrogen and progesterone. Hereby, this research made use of epidural anesthesia alone and epidural anesthesia combined with spinal anesthesia for 60 cases of painless labor in Cangzhou Hospital of Integrated TCM-WM·HEBEI, aiming at comparing their anesthesia effect and application value.

## Material and methods

### Patient clinical data

A total of 60 primiparas who had painless labor in Cangzhou Hospital of Integrated TCM-WM·HEBEI from June 2018 to January 2020 were selected as the research subjects. The maternal age was 21 to 32 years old, and the average age was (27.43 ± 2.62) years old. The authors divided them into either Spinal & Continuous epidural anesthesia group (n = 30) or the Continuous epidural anesthesia group (n = 30). Varying anesthesia methods were adopted respectively. The medical Ethics Committee had sanctioned this study, and all the pregnant women had been informed and submitted consent forms.

### Inclusion and exclusion criteria

Inclusion criteria: (1) Primiparas who had painless labor in Cangzhou Hospital of Integrated TCM-WM·HEBEI; (2) Primiparas aged ≥ 20 years; (3) Primiparas had complete clinical data and cooperated with the treatment; (4) No history of allergies to the anesthetic drugs used in this operation.

Exclusion criteria: (1) Pregnant women with immunodeficiency, infection or inflammatory diseases; (2) Pregnant women with severe organ dysfunction; (3) Pregnant women with mental illness.

### Research methods

(1) Anesthesia methods

Pregnant women in the Spinal & Continuous epidural anesthesia group were treated with spinal anesthesia combined with continuous epidural anesthesia. The anesthesiologist performed a puncture at the site of L2ཞL3 or L3ཞL4, and injected 5 mg of fentanyl into the subarachnoid space. An epidural catheter was inserted about 4 cm into the epidural space. 10∼15 min after the injection, the authors connected the self-controlled epidural analgesia pump to the epidural catheter. Using a 60∼ 65 mL mixture of 75 mg Naropin (AstraZeneca AB, H2010010550) and 50 μg fentanyl, the authors performed continuous infusion of 2 mL/h, added 5 mL/15‒20 min as needed, and stopped the infusion when the cervix was fully opened.

Pregnant women in the Continuous epidural anesthesia group were given continuous epidural anesthesia. The anesthesiologist performed a puncture at the site of L2ཞL3 or L3ཞL4, and placed the epidural catheter. After fixation, about 9 mL of 0.1% ropivacaine (Yichang Humanwell Pharmaceutical Co., Ltd., H20103636) and 30 µg of fentanyl (Jiangsu Nhua Pharma. Corporation, H20143315) were injected. Half an hour later, the pregnant women were tested for pain assessment and sensory and motor block. The authors connected the PCA pump and injected 60 mL of 0.1% ropivacaine and 30 µg of fentanyl to run at a rate of 6‒8 mL, and adjusted the release rate according to the pain of the parturient. The infusion was stopped when the cervix was fully opened.

(2) Serum sample collection

The authors collected 6 mL of peripheral venous blood from two groups of pregnant women while entering the operating room (T1), 0h (T2) and 24h (T3) after surgery, and the blood sample was left for 30 minutes. After the whole blood coagulated naturally and separated the serum, centrifugation was conducted at about 1000‒2000g at 4°C for 10 minutes to acquire the supernatant.

(3) Serum estrogen and progesterone detection

Using an automatic immunoassay analyzer (ORGENTEC, Germany, ORG 300), Progesterone (P), Luteinizing Hormone (LH), Follicle Stimulating Estrogen (FSH) and Estradiol (E2) levels were tested, and the operation was performed in stringent compliance with the instrument instructions.

(4) Inflammatory factor detection:

Serum TNF-α and IL-6 levels were assayed utilizing an ELISA kit (Beijing Jianping Jiuxing Biotechnology Co., Ltd.)

#### Observation indicators

The authors compared the serum estrogen and progesterone levels, the expression of inflammatory factors, the anesthetic effect, and the occurrence of postoperative complications of pregnant women, as well as the physical health of the newborns at different time periods of surgery.

(1) Serum estrogen and progesterone-related indicators of pregnant women

Serum P, LH, FSH, and E2 levels.

(2) Anesthesia effect and pain status of pregnant women

The anesthesia impact mainly included the onset and duration of sensory block and motor block. Furthermore, VAS [13] was adopted for assessing the pain condition of pregnant women before and after surgery. The total score was 0‒10 points. The lower the score, the lower the pain degree.

(3) Postpartum anxiety and depression of pregnant women and the physical condition of newborns 1 to 5 minutes after birth

The authors compared the anxiety and depression of the parturient 24 hours postpartum by Self-Rating Anxiety Scale (SAS) and Self-Rating Depression Scale (SDS). [14] The score was 0‒100 points. The higher the score, the more serious the anxiety and depression. The Apgar score [15] was used for comparing the physical condition of the newborn 1ཞ5 min after birth. The score was 0‒10 points. The lower the score, the worse the newborn's health.

(4) Inflammatory factors

The authors compared the serum TNF-α and IL-6 levels of the two groups of pregnant women, so as to observe the postoperative inflammatory activity.

(5) Incidence of postoperative complications in the two groups of pregnant women.

This study follows the STROBE statement.

### Statistical analysis

Statistical analysis was performed using the SPSS 25.0 software package, where measurements were expressed as mean ± standard deviation (x ± s). The *t*-test was used for intra-group comparisons. Enumeration data were expressed as percentages (%), and X^2^ test was used for comparison between groups. Differences were considered statistically significant at p < 0.05. GradpadPrism 7.0 software package was used for data visualization.

## Results

### Comparison of general information

**Fig. 1:**
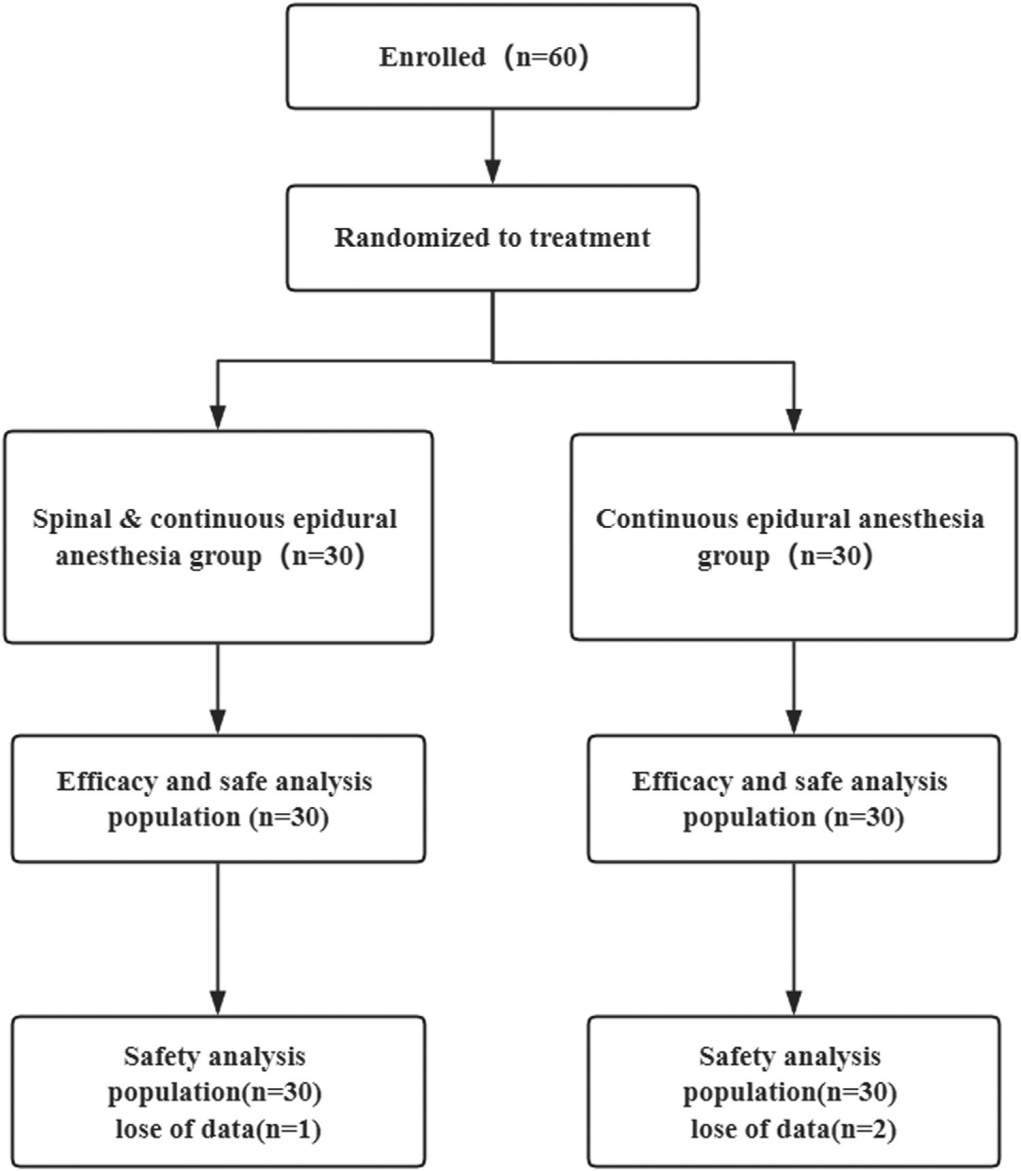
Research processes.

A total of 60 pregnant women were recruited for current work, and they were divided into two groups in accordance with the principle of voluntariness. No statistical significance was revealed between the two groups of pregnant women's age, BMI, pregnancy period and other general information (p > 0.05), and they were comparable. The patients were followed up for survival. There were 1 and 2 lost data in the Spinal & Continuous epidural anesthesia group and Continuous epidural anesthesia group, separately. See Table 1, Fig. 1.

**Table 1:** General information of the two groups of patients.

**Group**	**Spinal & Continuous epidural anesthesia** **(n = 30)**	**Continuous epidural anesthesia** **(n = 30)**	**T**	**p**
Mean age (years)	27.15 ± 2.49	28.24 ± 2.83	0.641	0.407
BMI (kg/m^2^)	23.16 ± 1.07	23.49 ± 1.09	0.623	0.418
Pregnancy (d)	278.51 ± 5.48	279.34 ± 5.59	0.583	0.526

BMI, Body Mass Index.

### Serum estrogen and progesterone related indicators

**Fig. 2:**
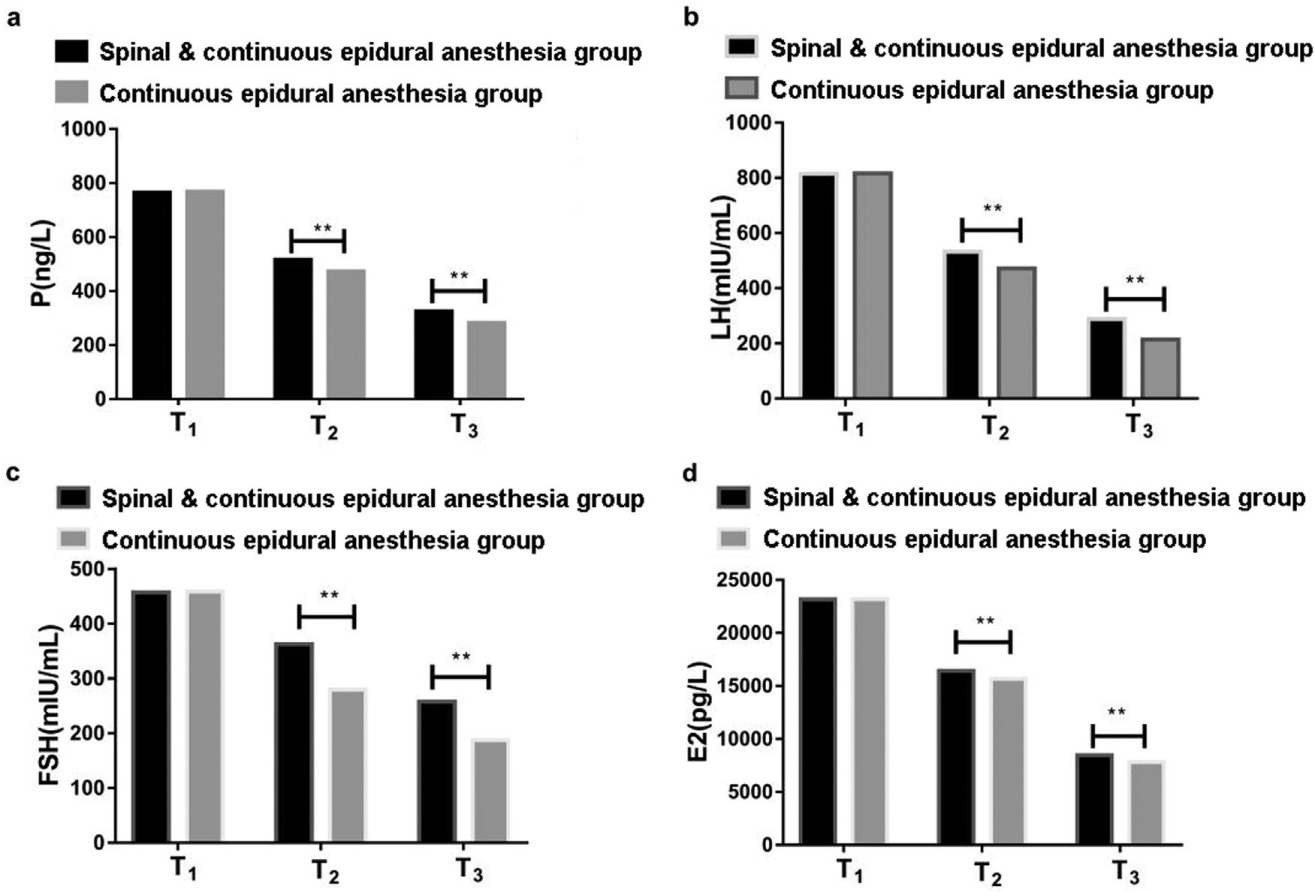
Comparison of serum estrogen and progesterone expressions. (a) Progesterone (P), (b) Luteinizing Hormone (LH), (c) Follicle Stimulating estrogen (FSH), (d) Estradiol (E2), entering the operating room (T1), 0h (T2) and 24h (T3) after surgery.

During T1, the differences in serum P, LH, FSH and E2 levels between the two groups of pregnant women were not statistically significant (p > 0.05). During T2 and T3, the serum P and LH levels of the two groups of pregnant women declined considerably, while the serum FSH and E2 levels elevated substantially, and the decline/rise of pregnant women in Continuous epidural anesthesia group was significantly higher than that in Spinal & Continuous epidural anesthesia group (p < 0.05). See Table 2, Fig. 2.

**Table 2:** Serum estrogen and progesterone related indicators (x ± s).

**Indicator**	**Time**	**Spinal & Continuous epidural anesthesia group** **(n = 30)**	**Continuous epidural anesthesia group** **(n = 30)**	**T**	**p**
P (ng/L)	T_1_	765.52 ± 124.17	768.14 ± 125.73	0.673	0.426
	T_2_	516.43 ± 72.95	472.85 ± 63.12	6.325	0.001
	T_3_	325.61 ± 46.72	281.74 ± 39.35	6.624	0.001
LH (miu/mL)	T_1_	814.62 ± 131.53	816.47 ± 128.75	0.572	0.541
	T_2_	531.74 ± 84.62	472.38 ± 79.03	6.414	0.001
	T_3_	287.82 ± 54.03	213.74 ± 39.46	6.254	0.001
FSH (miu/mL)	T_1_	457.23 ± 81.42	459.05 ± 76.84	0.684	0.413
	T_2_	362.59 ± 61.59	279.64 ± 48.36	6.752	0.001
	T_3_	257.52 ± 35.49	187.69 ± 27.48	6.594	0.001
E2 (pg/L)	T_1_	23183.52 ± 1219.63	23189.41 ± 1224.17	0.574	0.513
	T_2_	16429.41 ± 974.26	15672.59 ± 913.25	6.532	0.001
	T_3_	8464.28 ± 531.07	7824.31 ± 514.19	6.746	0.001

### Comparison of the anesthesia effect

**Fig. 3:**
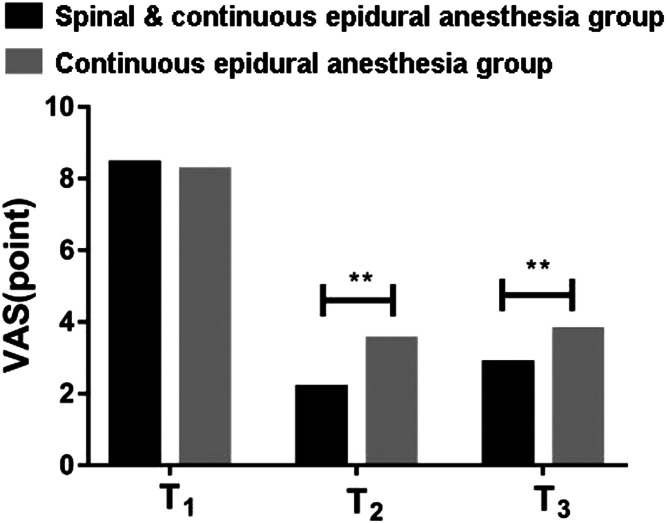
Comparison of the Visual Analogue Scale (VAS) pain scores 24 hours after surgery.

Compared with pregnant women in the Continuous epidural anesthesia group, pregnant women in the Spinal & Continuous epidural anesthesia group harbored shorter onset and longer duration of sensory block and motor block (p < 0.05). See Table 3. During T1, the difference in VAS scores of the two groups of pregnant women was not statistically significant (p > 0.05). During T2 and T3, the VAS scores of pregnant women in the Spinal & Continuous epidural anesthesia group were lower than those in the Continuous epidural anesthesia group (p < 0.05). See Fig. 3.

**Table 3:** Comparison of anesthesia effects (x ± s, min).

**Group**		**Spinal & Continuous epidural anestesia** **(n = 30)**	**Continuous epidural anesthesia** **(n = 30)**	**T**	**p**
Sensory block	Onset	8.74 ± 1.62	11.53 ± 2.47	5.732	0.003
	Duration	386.92 ± 25.64	342.51 ± 21.63	6.349	0.001
Motor block	Onset	11.34 ± 2.26	14.73 ± 2.61	5.268	0.005
	Duration	302.64 ± 17.59	283.54 ± 14.41	5.741	0.004

### Comparison of the postpartum anxiety and depression of pregnant women and the physical condition of the newborns 1- to 5-minutes after birth

**Fig. 4:**
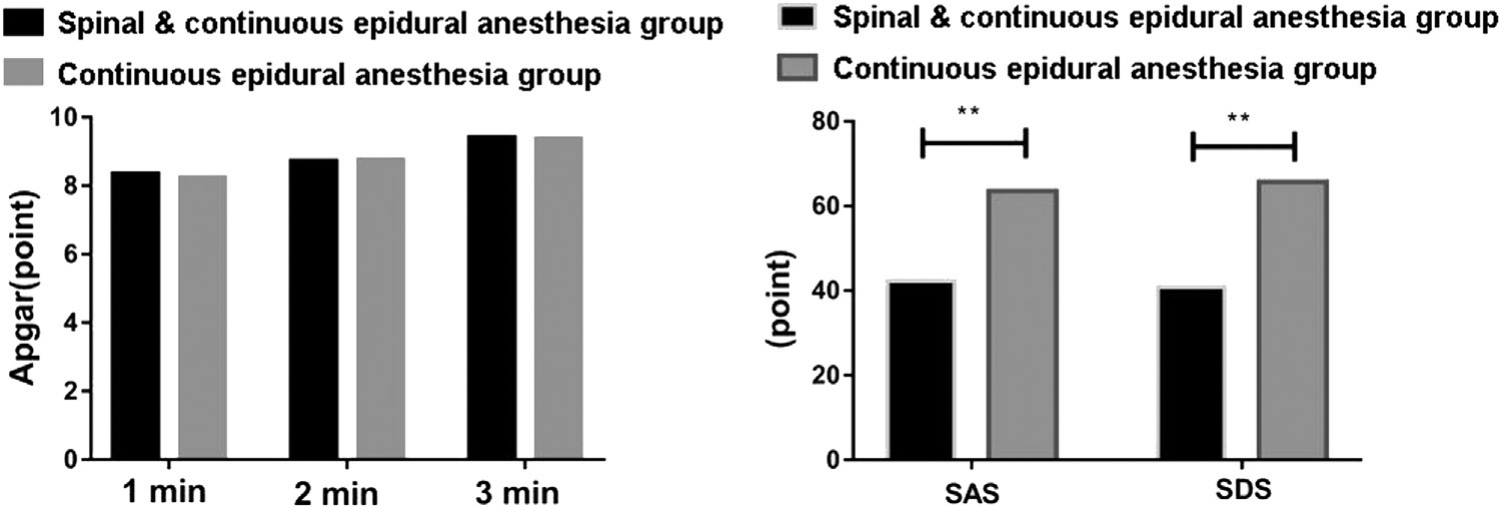
Comparison of the postpartum SAS, SDS and Apgar scores. The left was the comparison of the Apgar scores of newborns at 1-min, 3-min and 5-min after birth. The right was the comparison of Self-Rating Anxiety Scale (SAS) and Self-rating Depression Scale (SDS) scores of pregnant women.

No statistical significance was unveiled in the Apgar scores of the two groups of newborns at 1-min, 3-min, and 5-min after birth (p > 0.05). SAS and SDS scores of the pregnant women in the Spinal & Continuous epidural anesthesia group were brilliantly lower than those in the Continuous epidural anesthesia group (p < 0.05). See Table 4, Fig. 4.

**Table 4:** Comparison of the postpartum SAS and SDS of pregnant women and the Apgar scores of the newborns after birth (x ± s, points).

**Group**		**Spinal & Continuous epidural anesthesia** **(n = 30)**	**Continuous epidural anesthesia** **(n = 30)**	**T**	**p**
Apgar	1 min	8.35 ± 0.31	8.24 ± 0.37	0.673	0.425
	3 min	8.72 ± 0.35	8.75 ± 0.34	0.567	0.513
	5 min	9.42 ± 0.32	9.37 ± 0.30	0.623	0.451
SAS		42.37 ± 4.15	63.58 ± 7.26	6.738	0.001
SDS		40.92 ± 4.26	65.73 ± 7.45	6.417	0.001

### Comparison of inflammatory factor levels

**Fig. 5:**
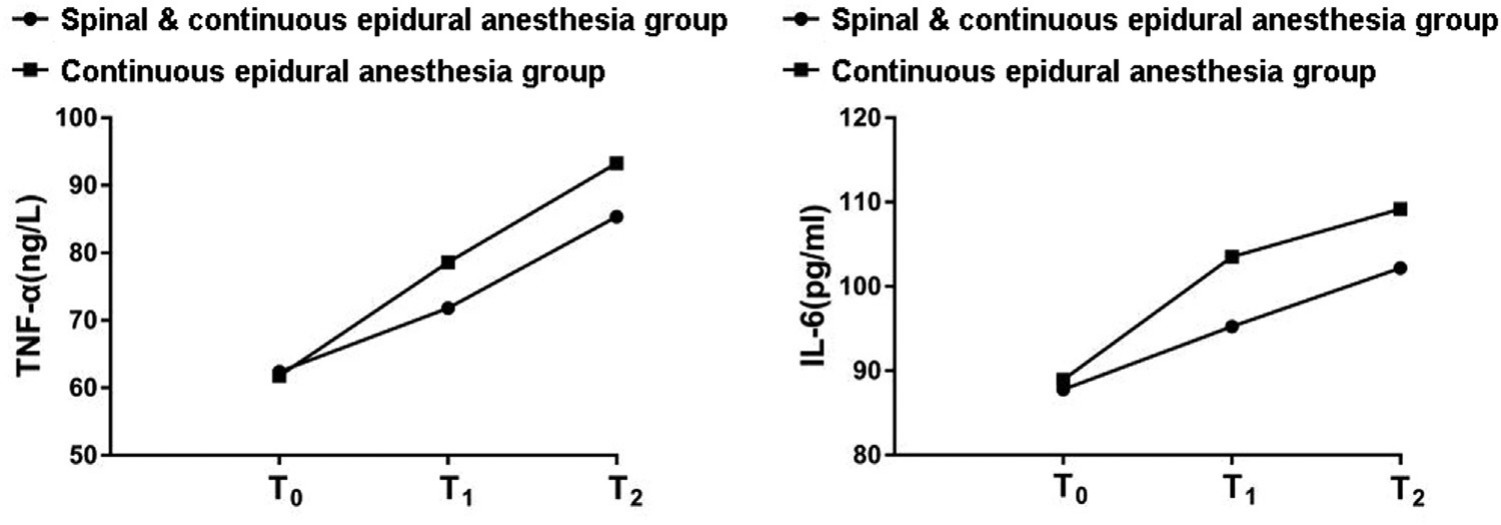
Comparison of the expression of postpartum inflammation indicators. ELISA kit was used to detect inflammatory cytokines. The left was a comparison of serum Tumor Necrosis Factor-α (TNF-α) expression in the two groups of pregnant women, and the right was a comparison of the serum Interleukin-6 (IL-6) expression of the two groups.

During T1, no significant difference was unmasked in serum TNF-α and IL-6 levels between the two groups of pregnant women (p > 0.05). During T2 and T3, the serum TNF-α and IL-6 levels of pregnant women in the Spinal & Continuous epidural anesthesia group were significantly lower than those of the Continuous epidural anesthesia group (p < 0.05). See Table 5, Fig. 5.

**Table 5:** Comparison of serum TNF-α and IL-6 levels (x ± s, ng/L).

**Group**		**Spinal & Continuous epidural anestesia** **(n = 30)**	**Continuous epidural anesthesia** **(n = 30)**	**T**	**p**
TNF-α	T_1_	62.41 ± 2.05	61.79 ± 2.03	0.546	0.512
	T_2_	71.82 ± 2.64^a^	78.59 ± 2.83^a^	5.575	0.002
	T_3_	85.37 ± 3.15^a^	93.28 ± 3.61^a^	0.683	0.412
IL-6	T_1_	87.79 ± 3.15	88.94 ± 3.16	0.661	0.423
	T_2_	95.26 ± 4.28^a^	103.51 ± 4.75^a^	5.784	0.002
	T_3_	102.17 ± 4.51^a^	109.17 ± 5.13^a^	0.569	0.525

Compared with T_0_

a p < 0.05.

### The incidence of postoperative complications

**Fig. 6:**
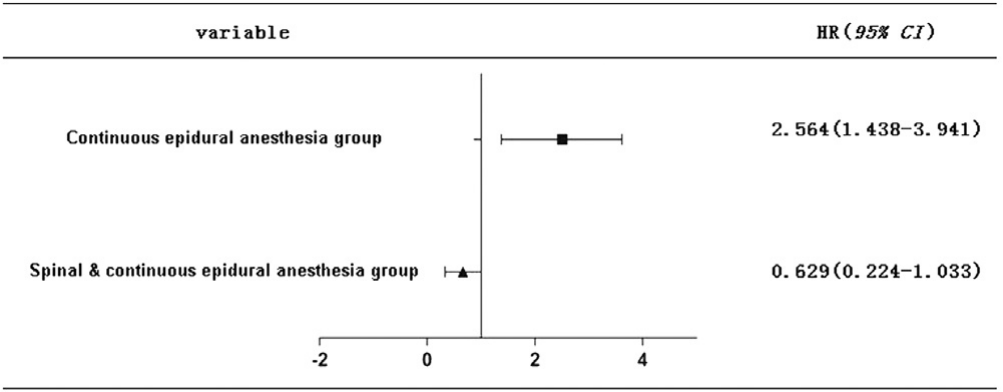
Comparison of the incidence of postoperative complications. The postoperative complications rate of pregnant women in Continuous epidural anesthesia group was higher than that in Spinal & Continuous epidural anesthesia group.

The total incidence of pregnant women in the Spinal & Continuous epidural anesthesia group was saliently lower than that in the Continuous epidural anesthesia group, with statistical significance (p < 0.05). See Table 6, Fig. 6. Compared with continuous epidural anesthesia, spinal anesthesia combined with continuous epidural anesthesia could manifestly reduce the incidence of postpartum complications in pregnant women who received painless labor.

**Table 6:** Comparison of postoperative complications [n, (%)].

**Group**	**Spinal & Continuous epidural anestesia** **(n = 30)**	**Continuous epidural anesthesia** **(n = 30)**	**X^2^**	**p**
Hypotension	1	1	‒	‒
Nausea, vomiting	1	2	‒	‒
Headache	0	2	‒	‒
Nerve damage	0	1	‒	‒
Postpartum hemorrhage	0	1	‒	‒
Total number	2	8	‒	‒
Total incidence (%)	6.67	23.33	6.136	0.001

## Discussion

With the advancement of the medical level and the development of anesthesiology, more and more women are going into labor without pain. [16] At present, the anesthesia used more often in surgery is intrathecal anesthesia. Intrathecal analgesia for labor and delivery is a method of anesthesia that can be administered independently according to the pain condition characterized by better anesthesia effect and high safety. [17] Intrathecal analgesic techniques, including single-needle spinal epidurals, standard epidurals, Combined Spinal Epidurals (CSE) and Dural Puncture Epidurals (DPE), are the most effective techniques for reducing labor pain. Nerve blocks for laboring patients provide reliable, rapid, high-quality analgesia with minimal serious side effects for the mother and fetus. [18]

Epidural anesthesia works by injecting anesthetic liquid into the epidural space and penetrating the intervertebral foramen after anesthetic diffusion. [19] Epidural anesthesia has the characteristics of a large dosage and slow onset of effect, moreover, excessive dosage may increase the incidence of adverse anesthesia. [18] Spinal anesthesia, namely subarachnoid anesthesia, principally blocks the nerve roots by injecting anesthetic liquid into the subarachnoid space through the lumbar intervertebral space. [20] The amount of spinal anesthesia is less, about 20% of the amount of epidural anesthesia, but due to the higher anesthesia site, it may cause head discomfort and other adverse reactions. [21]

The rapid changes of related factors in the body after delivery will cause changes in neurotransmitter secretion and abnormal brain excitability, which is the biological basis of postpartum depression. [22] In this paper, serum P, LH, FSH, and E2 levels in Spinal & Continuous epidural anesthesia group during and after delivery were significantly lower than those in Continuous epidural anesthesia group, and their postpartum SAS and SDS scores were also lower than those in Continuous epidural anesthesia group, indicating that the combination of spinal anesthesia and continuous epidural anesthesia can probably attenuate the psychological impacts of postpartum anxiety and depression of pregnant women by improving the rapid decline of serum estrogen and progesterone indicators in pregnant women with painless labor. Spinal anesthesia combined with continuous epidural anesthesia could dwindle the amount of anesthesia in the same location, alleviate the impact on the nervous system of the same location, and finally adjust the abnormal expression of postpartum serum estrogen and progesterone to a certain extent.

In this work, the pregnant women of the Spinal & Continuous epidural anesthesia group owned preeminently better anesthesia effects for the reason that their anesthesia onset time was prominently shorter, and the anesthesia duration was observably longer than those of the Continuous epidural anesthesia group. Wang H et al. [23] corroborated that due to different injection sites, the onset time of spinal anesthesia was noticeably shorter than that of epidural anesthesia, which was consistent with the results of this study. The combination of spinal anesthesia and epidural anesthesia can exert the anesthesia impact on different body parts and ways, which is conducive to enhancing the anesthesia effect and lengthening the anesthesia duration. Additionally, VAS scores of pregnant women in the Spinal & Continuous epidural anesthesia group were lower than those in the Continuous epidural anesthesia group during and after delivery, which also illustrated the superiority of the anesthesia effect of the Spinal & Continuous epidural anesthesia group to a certain extent. Apgar score is a prevalently utilized clinical index to assess the physical health of newborns within 1 to 5 minutes after birth. [15] In this research, no significant difference was disclosed in Apgar scores at 1-min, 3-min and 5-min after birth, suggesting that spinal anesthesia combined with epidural anesthesia and epidural anesthesia alone would not affect the physical health of newborns within 5-min after birth.

Clinical studies have substantiated that surgical trauma may elevate the level of inflammatory activity in the body and escalate the incidence of related complications. [24] TNF-α is a multi-directional cytokine with a two-way regulatory effect. When TNF-α is at a normal level, it possesses anti-tumor function. However, a pathological increase in TNF-α level will have a counterproductive effect, damage the body's immune function, intensify the level of inflammatory activity, and are tightly connected to the emergence and progress of multiple inflammatory or infectious diseases in the body. [25] As the main inflammatory factor, IL-6 takes part in the emergence and progression of diverse inflammatory activities in the body. [26] Pringle K G et al. [27] authenticated that after painless labor, the serum TNF-α and IL-6 levels of pregnant women with severe complications were sensibly higher than those of pregnant women without complications. In the present research, the postoperative serum TNF-α and IL-6 levels of pregnant women in the Spinal & Continuous epidural anesthesia group were notably higher than those in the Continuous epidural anesthesia group, implicating that spinal anesthesia combined with continuous epidural anesthesia potently reduced the levels of inflammatory factors that increased by surgery and governed the inflammatory activity in pregnant women receiving painless labor. The total incidence of postoperative complications was visibly lower in the Spinal & Continuous epidural anesthesia group than Continuous epidural anesthesia group, which further directly implied that spinal anesthesia combined with continuous epidural anesthesia harbors an outstanding impact on reducing the incidence of postoperative complications in pregnant women with painless labor, and ultimately promoting the recovery of their postpartum physical fitness.

In summary, in the painless labor of primiparas, compared with continuous epidural anesthesia, spinal anesthesia combined with continuous epidural anesthesia harbors a better anesthesia effect, efficaciously meliorating the pregnancy process and postpartum serum estrogen and progesterone levels, which can be extensively promoted and applied clinically. There are still some limitations in this work. For instance, this study only delved into the anesthesia regimen for primiparas. Hence, the authors can conduct more exploration of the anesthesia plan for multiparas and investigate of the relationship between serum estrogen and progesterone in future studies to provide more clinical information.

## Availability of data and materials

The datasets used and/or analyzed during the present study are available from the corresponding author upon reasonable request.

## Ethics statement

The present study was approved by the Ethics Committee of Cangzhou Hospital of Integrated TCM-WM·HEBEI (approval number: 201801CZ03) and written informed consent was provided by all patients prior to the study start. All procedures were performed in accordance with the ethical standards of the Institutional Review Board and The Declaration of Helsinki, and its later amendments or comparable ethical standards.

## Authors’ contributions

JunYan Liu and ChongLai Shi designed the research study. Dan Wang, XiaoDong Cui and LiLi Geng performed the research. JingJing Cui, DongMei Sun and Zhuo Yin provided help and advice on the experiments. Dan Wang, XiaoDong Cui and LiLi Geng analyzed the data. JunYan Liu and ChongLai Shi wrote the manuscript. JunYan Liu, ChongLai Shi and Zhuo Yin reviewed and edited the manuscript. All authors contributed to editorial changes in the manuscript. All authors read and approved the final manuscript.

## Funding

This study was supported by the General Project of Scientific Research Plan of Hebei Administration of Traditional Chinese Medicine (n 2023450).

## Declaration of competing interest

The authors declare no conflicts of interest.
